# A contribution to age determination of cheetahs (*Acinonyx jubatus*) based on radiographic analysis of the skull and postcranial morphology

**DOI:** 10.1371/journal.pone.0217999

**Published:** 2019-06-11

**Authors:** Martin J. Schmidt, Gerhard Steenkamp, Klaus Failing, Peter Caldwell, Robert M. Kirberger

**Affiliations:** 1 Department of Veterinary Clinical Sciences, Small Animal Clinic, Justus-Liebig-University, Frankfurter Strasse, Giessen, Germany; 2 Department of Companion Animal Clinical Studies, Faculty of Veterinary Science, Onderstepoort, South Africa; 3 Unit for Biomathematics and Data Processing, Faculty of Veterinary Medicine, Justus Liebig-University-Giessen, Giessen, Germany; 4 Old Chapel Veterinary Clinic, Villeria Pretoria, South Africa; Royal Veterinary College, UNITED KINGDOM

## Abstract

The aim of this retrospective cross-sectional study was to present comprehensive information about the age-dependent change of skeletal characteristics in captive cheetahs with known age and to assess the benefit of these variables for age estimation in this species. Radiographs of 162 known-age captive and semi-captive cheetahs were retrospectively examined and age-related changes of skull, axial and appendicular skeletal systems were documented. Metric and non-metric variables were used. These parameters were checked for the best correlation with age using a multiple stepwise regression analysis. An overview about the time frames, in which ossification centers appeared and physeal closure occurred is presented. Multiple stepwise regression analysis revealed the status of closure of the coronal suture, the maximum length of the frontal sinus, the condylobasal-, hard palate, and facial length are most significantly correlated with age. Together with the pulp size of the upper canine, these values can be used for an age approximation in cheetahs.

## Introduction

The cheetah (*Acinonyx jubatus*) is one of the most endangered carnivoran species with less than 15.000 animals dispersed over Africa [1]. Given their conservation needs cheetah populations are intensively monitored [1]. A prerequisite for many conservation investigations is a reliable method for age determination in order to calculate demographics [2–5]. Age is also a critical variable in epidemiological investigations [6] as disease susceptibility and prevalence may vary among age groups.

Felids can be aged on the basis of various morphological characteristics. Tooth wear, gum recession and the dimensions of canine pulp cavity are used for age determination in lions, tigers, leopards, bobcats, and mountain lions [7–13]. The continued formation of dentine in a tooth increases with age, causing the pulp cavity to become smaller with time [13–16]. The radiographic determination of the pulp cavity size has been successfully used to age wild dogs [13] and felids [14–16]. The documentation of ontogenic skull changes is also useful for age determination in carnivorans. Established cranial landmarks are used in many scientific disciplines as reference points from which linear measurements can be taken to analyze physiological [17–24] and pathological growth trajectories [25, 26]. A variety of these landmarks and measurements have been well established in felids [27–29] and can be used for age determination [30–34]. With increased growth of the skull, there is a gradual obliteration of calvarial sutures that can be correlated with age in carnivorans [35–38] and in humans [39]. Another skull parameter that was utilized to predict the unknown age of humans [40] and other animals is the frontal sinus size [41, 42].

With maturity, growth rate of long bones decreases due to the reduced activity of the cartilage that gradually disappears resulting in complete fusion of various ossification centers and physes. Age estimation based on changes of the skeleton has been successfully used in many domestic species, including the dog and cat [43, 44]. The physes of the vertebrae ossify in a similar pattern and gradually close during ontogeny [45].

Overall, detailed investigations describing the timing of the above mentioned developmental changes in the cheetah have not been documented. Gross categorization of adult and immature individuals was proposed [46], but data useful for a more precise age determination are still lacking. The aim of this retrospective cross-sectional study was to document changes in skeletal characteristics, identify those variables that correlated best with age, and to integrate the results into a method for age estimation in captive cheetahs based on radiographic analysis.

## Materials and methods

The picture archiving and communication system (PACS) of the Onderstepoort Veterinary Academic Hospital, Faculty of Veterinary Science, University of Pretoria and a private veterinary wildlife clinic (Old Chapel Veterinary Clinic) were searched for digital radiographs of cheetahs. Only South African cheetahs of known age were included. King cheetahs were excluded. Age, weight and sex of cheetahs were recorded.

### Ethics statement

This study was approved by the University of Pretoria Animal Use and Care Ethics committee (No. REC 107–18) and the Faculty of Veterinary Science Research Ethics Committee (No. v001-19).

### Imaging technique

Radiographs were made with a variety of computed radiography systems (Kodak Point of Care CR360, Carestream Vita SE system and Fuji Capsula system). Exposure settings varied and depended on the age of the cheetah and body part examined. Orthogonal views included dorsovental (DV) and latero-lateral head views, ventrodorsal (VD) and latero-lateral vertebral column and mediolateral (ML and craniocaudal (CrCd) and dorsopalmar (plantar) (DPa, DPl) views of the appendicular skeleton. Radiographs were obtained under general anesthesia as part of routine annual health examinations, or of ill or traumatized cheetahs. Owner consent for use of clinical data was automatically obtained when owners agreed to the examination of the cheetahs. All data were anonymized.

### Image analysis

All radiographs were retrieved from the relevant PACS system and evaluated retrospectively by a board certified neurologist (MJS) and board certified radiologist (RMK), who were unaware of the cheetahs’ age or sex. Studies were evaluated with open source DICOM viewing software and window levels, window widths, and magnification were adjusted as needed in order to optimize visualization of anatomical detail.

Many radiographs were made under field conditions and all radiographs were thus assessed for adequate technical quality and for proper positioning. The rotation of the head and/or spine was graded from 0 (perfect positioning) to 5 (severely rotated). Radiographs were not considered eligible for the study if they were graded 4 or 5.

Not all body parts were radiographed in every cheetah and thus sample sizes varied for each collected measurement. All measurements/interpretations were made independently by the two observers to determine interobserver variability. Data were averaged between the two observers for all analyses.

### Skull measurements

**Fig 1:**
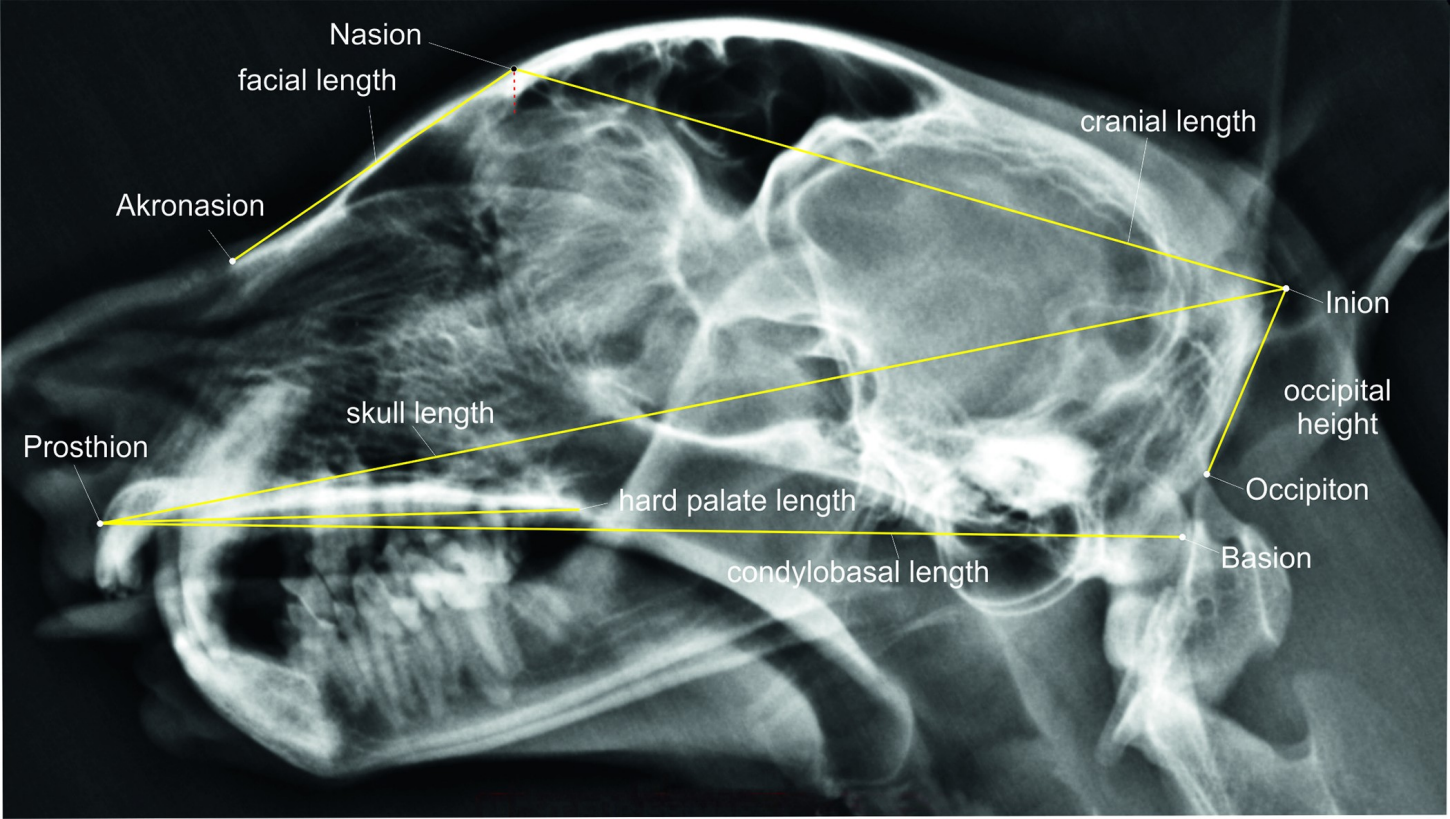
Craniometric measurements on a cheetah skull as seen from the lateral view. Laterolateral skull radiograph of a cheetah demonstrating the linear cranial measurements.

The following cranial points, as described in other studies [30–34], were identified on the cheetah skulls:

**Akronasion:** Most rostral end of the nasal bone (Fig 1).

**Basion:** Caudal surface of the occipital condyles (Fig 1).

**Fig 2:**
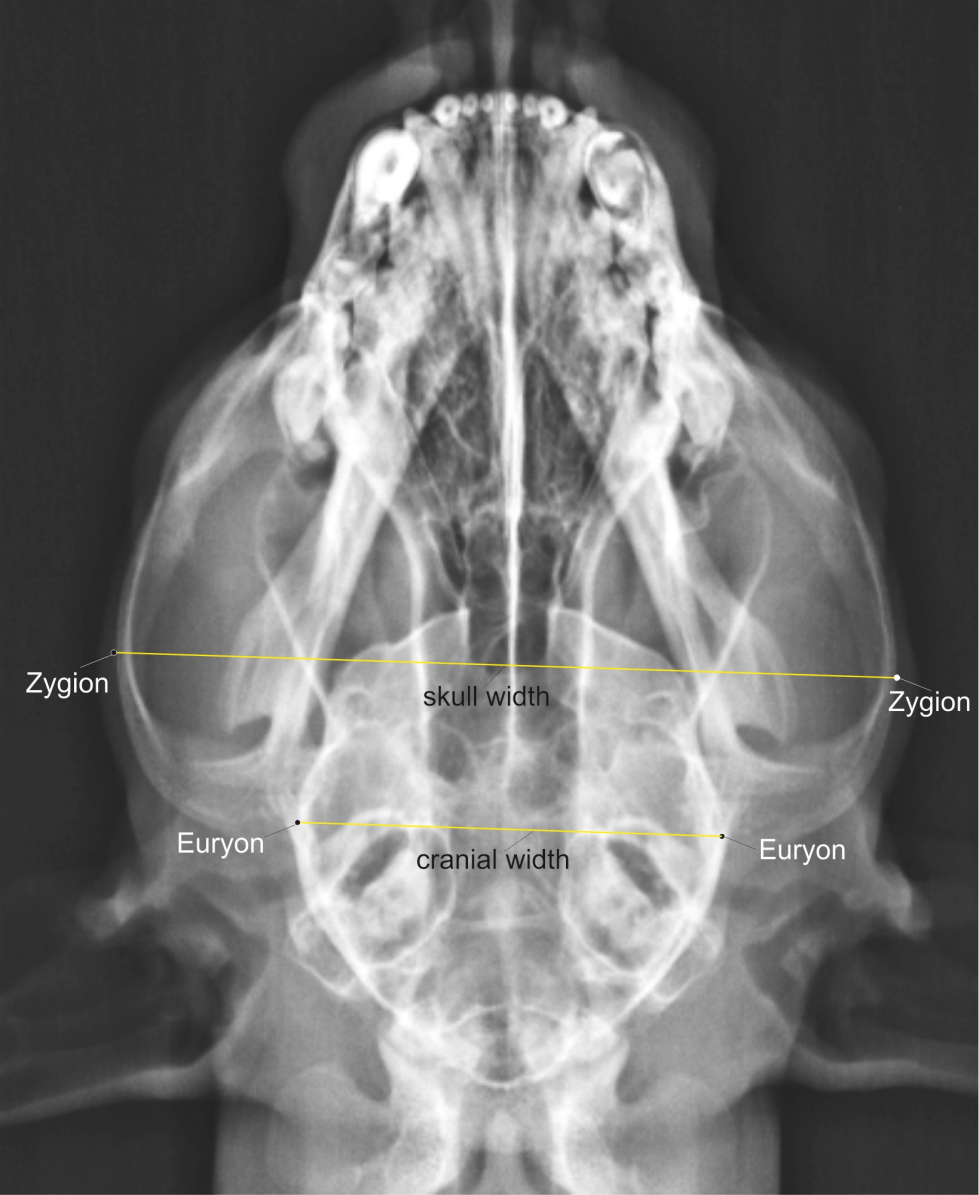
Craniometric measurements on a cheetah skull as seen from the dorsal view. Ventrodorsal skull radiograph of a cheetah demonstrating the linear cranial measurements.

**Euryon**: The most lateral point on the parietal bone seen in a dorsoventral view (Fig 2).

**Inion**: Most caudodorsal midline point on the external occipital protuberance (Fig 1).

**Nasion**: Junction on the midline of the left and right naso-frontal sutures. In latarolateral radiographs the point is dorsal to the rostral end of the frontal sinus (Fig 1).

**Occipiton**: Most dorsal point of the foramen magnum (Fig 1).

**Prosthion**: The point of the upper alveolar process that projects most rostrally in the midline (Fig 1).

**Zygion**: The most lateral point on the zygomatic arches seen in a dorsoventral view (Fig 2).

Based on the above landmarks the following linear measurements were taken [28–30] (Fig 1):

**Cranial length**: Distance from inion to nasion (Fig 1).

**Cranial width:** Distance from euryon to euryon (Fig 2).

**Condylobasal length**: Distance from the prosthion to the basion (Fig 1).

**Facial length**: Distance from the nasion to the akronasion (Fig 1).

**Hard palate length:** Distance from prosthion to the caudal end of the hard palate (Fig 1).

**Occipital height**: Distance from occipiton to inion (Fig 2).

**Skull length:** Distance inion to prosthion (Fig 1).

**Skull width:** Distance from zygion to zygion (Fig 2)

#### Dimensions of the frontal sinus

**Fig 3:**
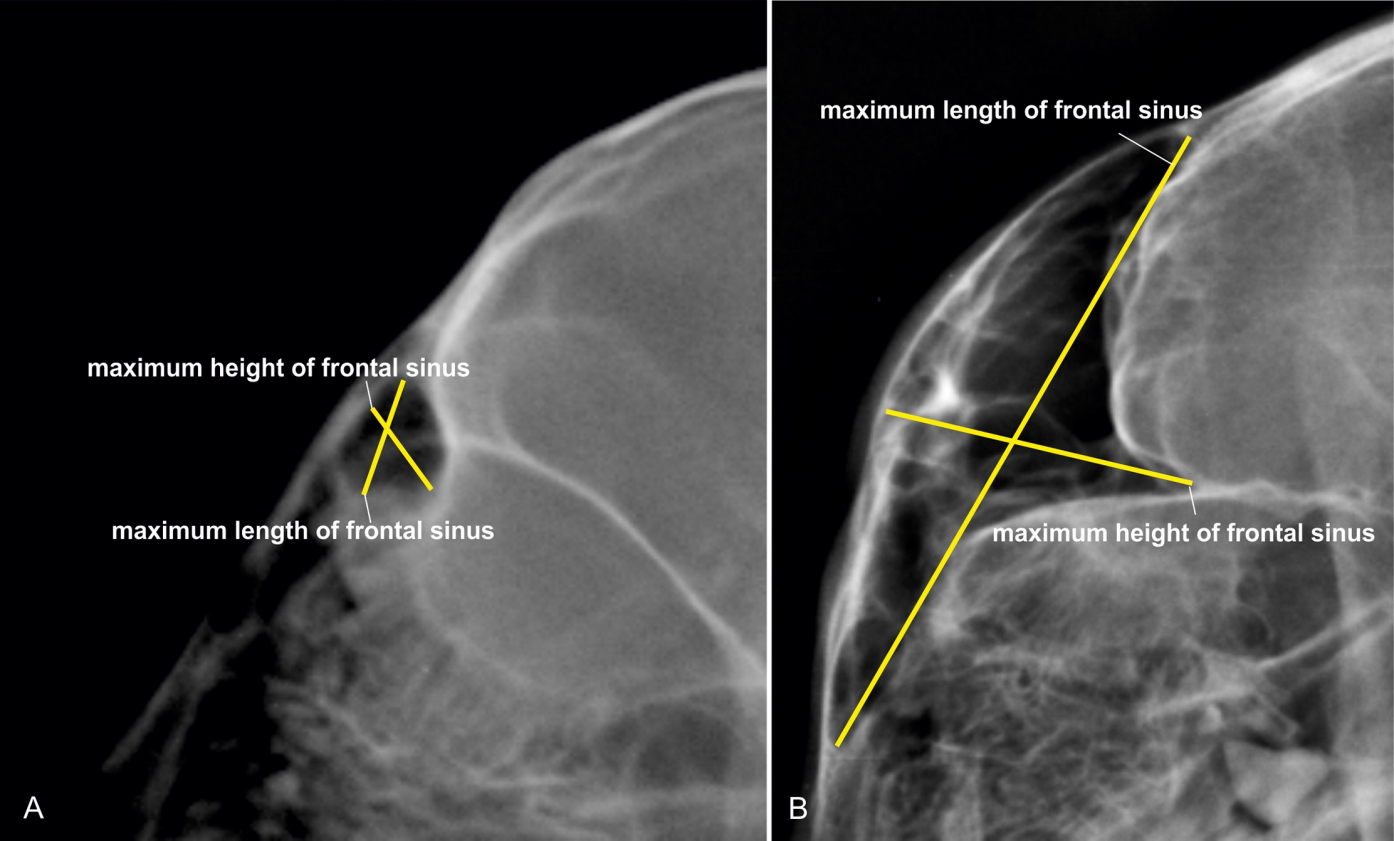
Measurement of the frontal sinus in cheetahs. Laterolateral skull radiographs of a (A) 6 months and (B) 72 months old cheetah demonstrating the measurement of the frontal sinus dimensions. The frontal sinus index in A was 0.48 and in B 0.32.

During this investigation we have noticed an ongoing expansion of the frontal sinuses in growing cheetahs. The change of the frontal sinus dimensions could potentially be used for a species-specific ageing method and was added to the model. The dimensions of the frontal sinus were determined from the latero-lateral view. The maximum length and height of the frontal sinus was measured, and then put into relation as a frontal sinus index (FSI = length/height) (Fig 3).

#### Pulp cavity

**Fig 4:**
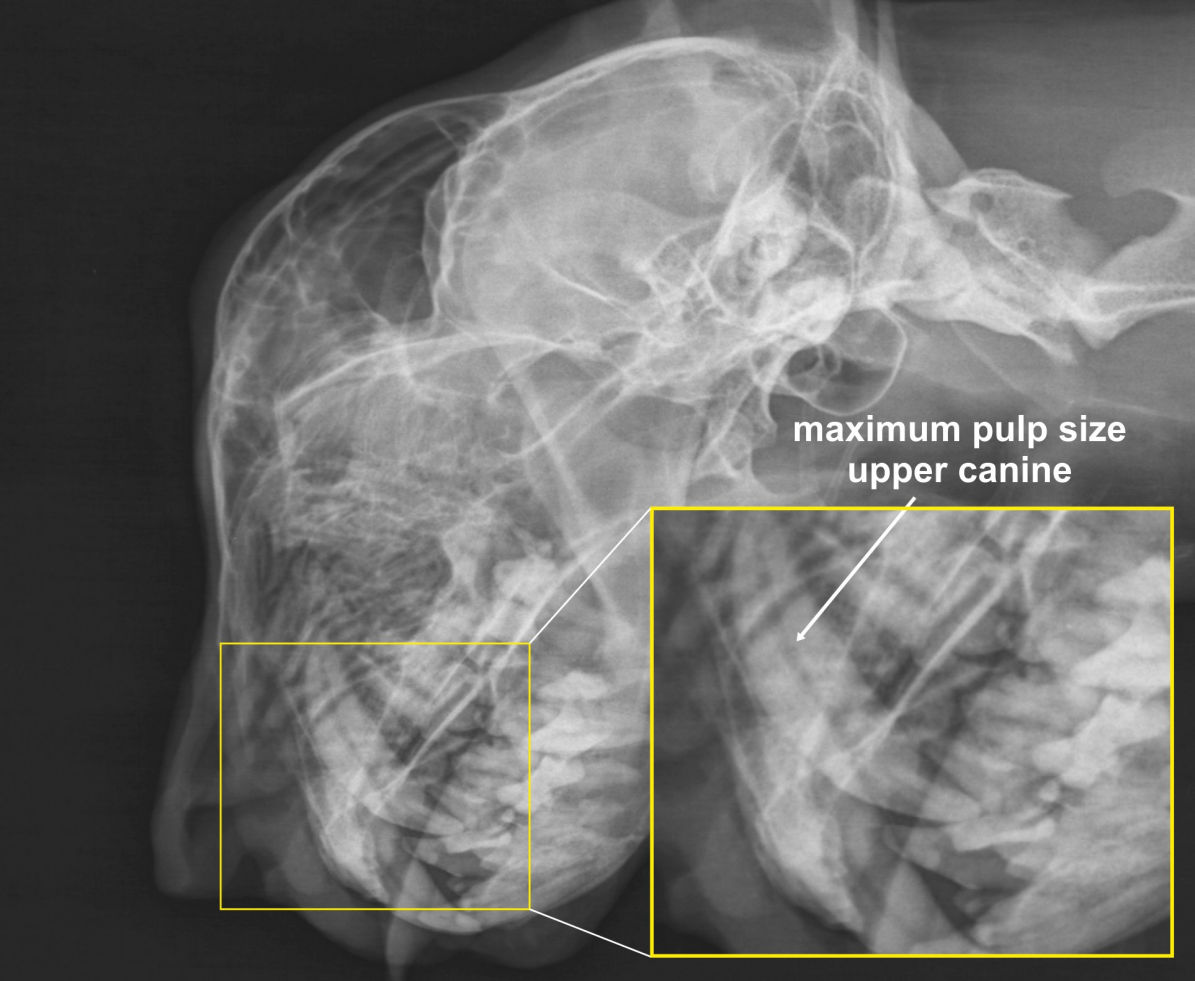
Pulp size measurement in a cheetah. Laterolateral skull radiograph of a cheetah demonstrating the determination of the pulp size in the upper canine.

The maximum rostrocaudal diameter of the radiolucent pulp cavity was measured in the best visualized upper canine on the latero-lateral view (Fig 4) [15, 16].

#### Ossification status of the coronal suture

**Fig 5:**
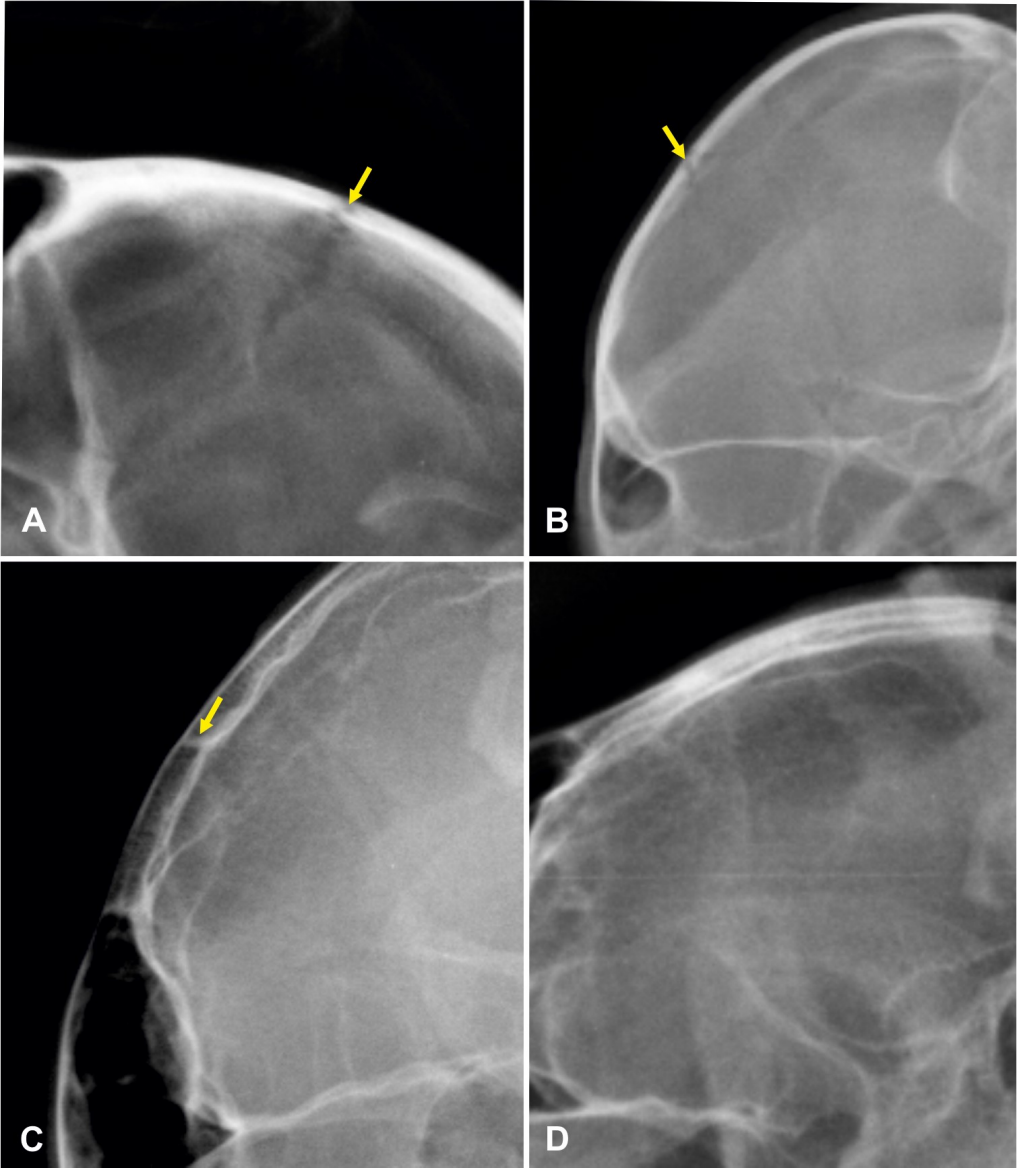
Gradual obliteration of the coronal suture in cheetahs. Laterolateral skull radiographs of different age cheetahs demonstrating the gradual obliteration of the coronal suture. A is 3.2 months old and graded 0; B is 10 months old and is graded 1; C is 18 months old and is graded 2 and D is 149 months old and is graded 3.

The open or closed status of the coronal (fronto-parietal) suture was graded as follows: Obvious serrated lucent lines between the frontal and parietal bones was graded as 0; a smaller but visible smooth lucent line as 1; and a suture scar, seen as a radiopaque line between the calvarial bone marrow cavity, as 2. An absent (closed) suture was graded as 3 (Fig 5) [45].

#### Vertebral column measurements

**Fig 6:**
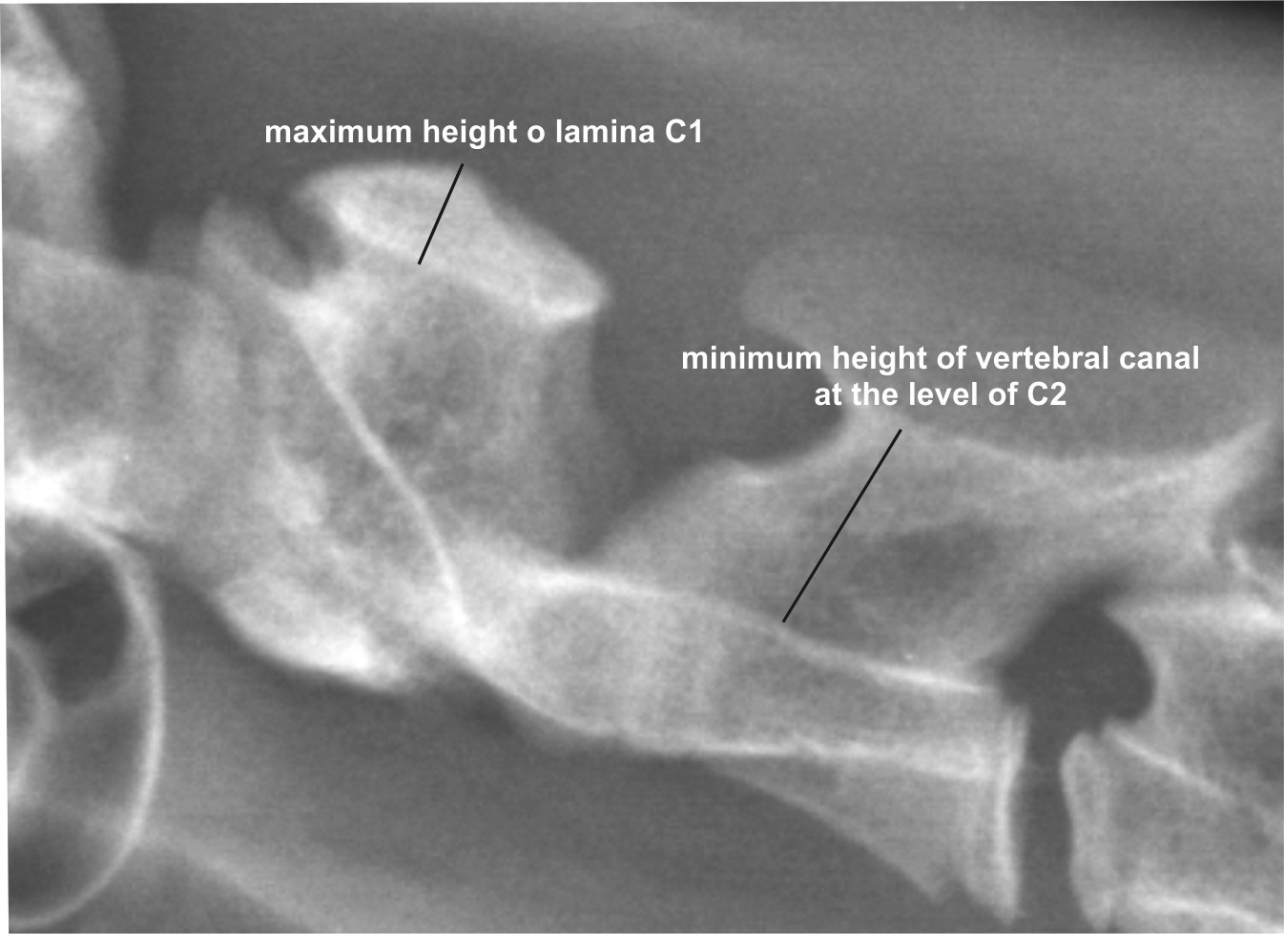
Measurements on atlas and axis of a cheetah. Laterolateral radiographs of the cranial cervical spine of a cheetah demonstrating the measurement of the maximal height of the lamina of the atlas and the minimal diameter of the vertebral canal on the level of the axis.

Atlas (C1): The maximum height of the lamina was measured (Fig 6).

**Fig 7:**
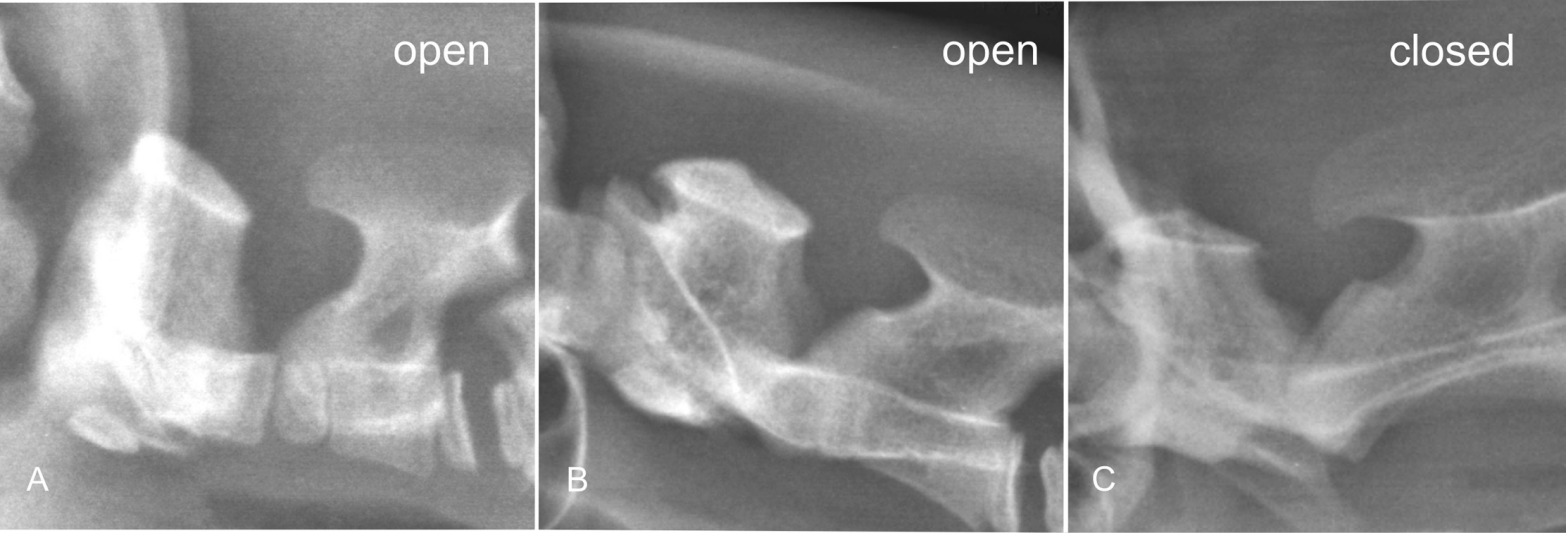
Assessment of the ossification of the dens axis in cheetahs. Laterolateral radiograph of the upper cervical spine of different age cheetahs demonstrating the gradual ossification of the dens axis. A is 3.2 months old, B is 6 months old, and C is 42 months old and the dens is completely fused.

Axis (C2): The minimum height of the vertebral canal was measured perpendicularly from the vertebral canal floor to the closest edge of the lamina (Fig 6). The ossification status of the intercentrum II of the axis was assessed as unossified, or if no physeal lines were visible, the intercentrum was assessed as ossified (Fig 7). Additionally the closure of the caudal C2 physis was evaluated.

**Fig 8:**
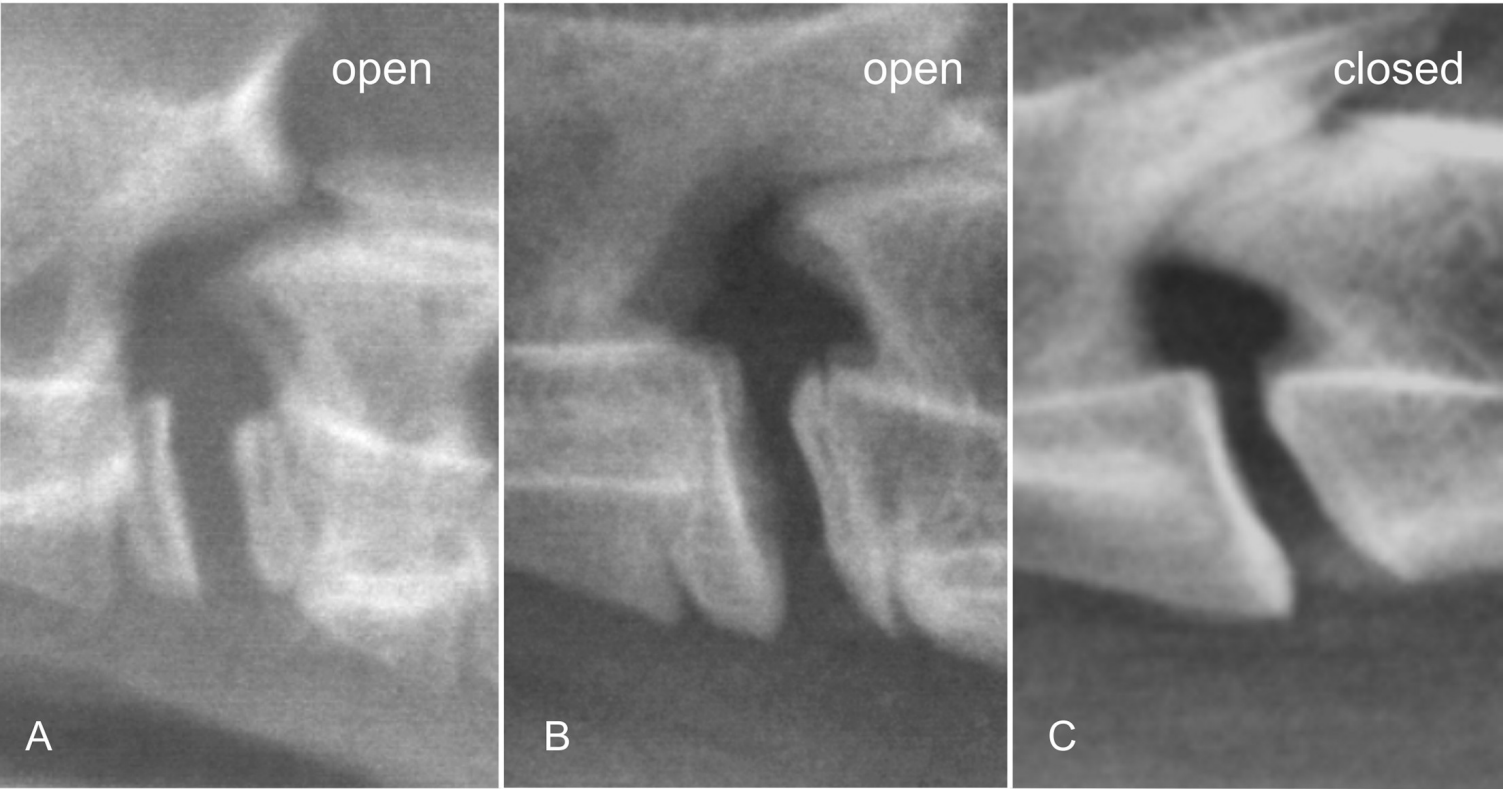
Laterolateral radiographs of the C4 –C5 cervical spine region of different age cheetahs demonstrating the gradual ossification of the cervical physes. A is 3.2 months old, B is 6 months old, and C is 42 months old.

Vertebral body physeal closure: The closure of the rostral and caudal physes of the fourth cervical (C4) and fifth lumbar (L5) vertebral bodies was evaluated as representative samples of spinal physeal fusion (Fig 8). All physes were graded as open or closed.

#### Appendicular skeleton measurements

**Fig 9:**
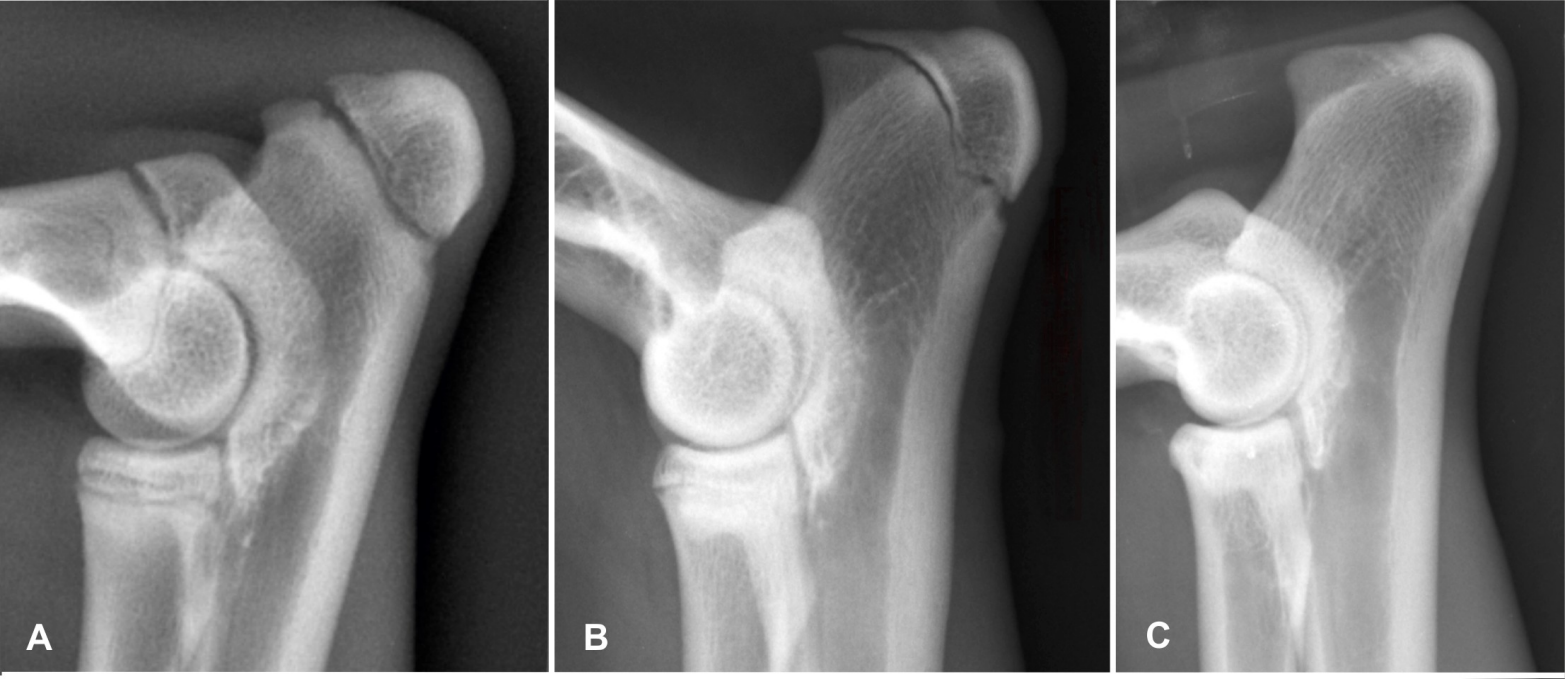
Gradual ossification of the olecranon physis. Medio-lateral elbow radiographs showing the gradual obliteration of the olecranon physis. A is 6 months old; B is 10 months old, and C is 24 months old. Note the presence of humerus and radius physes still in A.

Physeal closure and apophyseal ossification of long bones: The physis was assessed as open when a clear radiolucent separation between the epiphysis and metaphysis could be observed. The presence of a continuous physeal scar or a continuous medullary cavity between epi- and diaphysis was thus assessed as closed. The apophyses of the supraglenoid tubercle, major humerus tubercle, medial humeral epicondyle, olecranon tubercle, caudal accessory carpal bone, major trochanter of the femur, tibial crest and calcaneus tubercle were reported as unfused (0) or fused (1). In the unfused cases the percentage of physeal closure was subjectively assessed. An example of the assessment of the olecranon tubercle is given in Fig 9. As the clavicle and patella only start ossifying after birth their presence was determined to be incompletely or completely ossified.

### Statistical analysis

All statistical test procedures were performed using a commercially available software package Graph Pad Prism 4.0; Graph Pad Software Inc., San Diego, California. Graphical presentations were created using the same software.

First, the relationships between the measured variables and age were checked by correlation analysis. The correlation of the parameters with sex was tested on the variables by means of a one-way analysis of covariance using sex as the grouping variable. The relationships between the predictors and the parameter age were found to be non-linear in all cases showing a nearly exponential course. Therefore, a log transformation of age was performed to get a nearly linear relation to the other quantitative variables. Because in a few cases the result was not completely satisfactory, the regression equation was extended by a quadratic term to correct for the slight curvilinear course of the scatter plot. In the next step, data were analyzed with a stepwise multiple linear or quadratic regression analysis to identify a set of the most relevant variables that showed closest correlation with age. However, in this step the different number of available observations of these variables, due to missing values, had to be taken into consideration. In the first step of the analysis, each variable was considered separately for its predicting power to the age of the animal and the variable with the highest correlation (resp. coefficient of determination r^2^) was included into the model. After this, the remaining variables were tested for further improvement of the age estimation. F-statistics and multiple coefficients of determination (adjusted r^2^) as a measure for the goodness of fit of the regression equation were reported. The higher the F-value, the better the statistical significance. Repeating this step, regression coefficients with highest correlation with age were used to create an equation allowing to estimate the age of the animal in months.

To assess how accurate the model was, the predicted and observed log-transformed age were compared using a normal probability plot for the residuals. Residuals are estimates of the observational error obtained by subtracting the predicted age from the observed age. Additionally, the standard error of the log-age estimation was calculated by means of the multiple regression analysis.

In order to assess the interobserver variability for the measured predictor variables, the reproducibility of the measurements was determined using a Bland-Altman analysis, which considers the differences between the two observers for each parameter. The differences between the two measurements were then plotted against the average of the two measurements. Reproducibility was considered good, if 95% of the differences were within these two standard deviations [47]. P-values less than 0.05 were considered to be statistically significant. Interobserver differences for nominal data (physeal closure) were determined using kappa statistics [47].

## Results

### Animals

The majority of cheetahs came from the De Wildt Cheetah Research Centre near Pretoria and their farm, De Wildt Shingwetzi, near Bela Bela in the Limpopo province. The remainder came from small cheetah collections owned by lodges as tourist-attraction game farms. The animals were accommodated in varying sized fenced bushveld camps in small groups. Their diets varied, but would generally consist of meat and bones provided on six days of the week and often had commercially available mineral and vitamin supplements added. Data and radiographs from 162 cheetahs from between May 2010 and February 2016 were retrieved and reviewed with each having a variable number of radiographs made of a single or multiple anatomical regions. Fourty-six cheetahs were rejected either because of poor radiographic quality, poor positioning or due to suspected metabolic bone diseases (e.g. nutritional secondary hyperparathyroidism and rickets). Radiographs were evaluated from 109 anaesthetized cheetahs and from 6 post mortem partially dissected heads. There were 54 males and 57 females, whilst for four the sex was unknown. Their ages ranged from 1 month to 182 months (15 years). Twelve cheetahs were examined twice, and four animal three times all at different ages giving longitudinal data.

### Measurements and age estimation

The values of cephalometric and dental measurements as well as physeal closure for each individual cheetah are presented in S1 Table (supplemental material). Table 1 gives an overview of the time frames, in which ossification centers appeared and physeal closure occurred.

**Table 1:** Overview of the appearance of ossification centers and physeal closure in the cheetah.

Site	Approximate age at which ossification center appears/physeal closure starts	Approximate age at which physeal closure is completed
Accessory carpal apophysis	6 months	11–12 months
C2 caudal physis	3 months	12–36 months
C4 cranial physis	-	18 months
C4 caudal physis	3-4months	36 months
Calcaneus apophysis	8 months	62 months
Distal humerus physis	6–7 months	10–12 months
Distal radial physis	24 months	31 months
Distal radial physis	24 months	31 months
Distal femoral physis	7–8 months	31 months
Distal tibial physis	12 months	72 months
Distal ulnar physis	10 months	31 months
Femoral head physis	8 months	10 months
L5 caudal physis	2–3 months	20–30 months
L5 cranial physis	2–5 months	11 months
Major tubercle apohysis	5–6 months	18–21 months
Medial epicondyle apophysis	6–7 months	12 months
Metacarpal physis	-	10 months
Ossification of dens axis	1 month	11 months
Olecranon apophysis	2–8 months	18 months
Proximal humerus physis	5–6 months	18–31 months
Proximal radius	5–6 months	18 months
Proximal tibial physis	7–10 months	62 months
Patella visible	3–5 months	6.5–7 months
Supraglenoid apophysis	5 months	10–11 months
Trochanter major apophysis	6–7 months	31 months
Tibial crest apohysis	5–7 months	62–72 months

**Fig 10:**
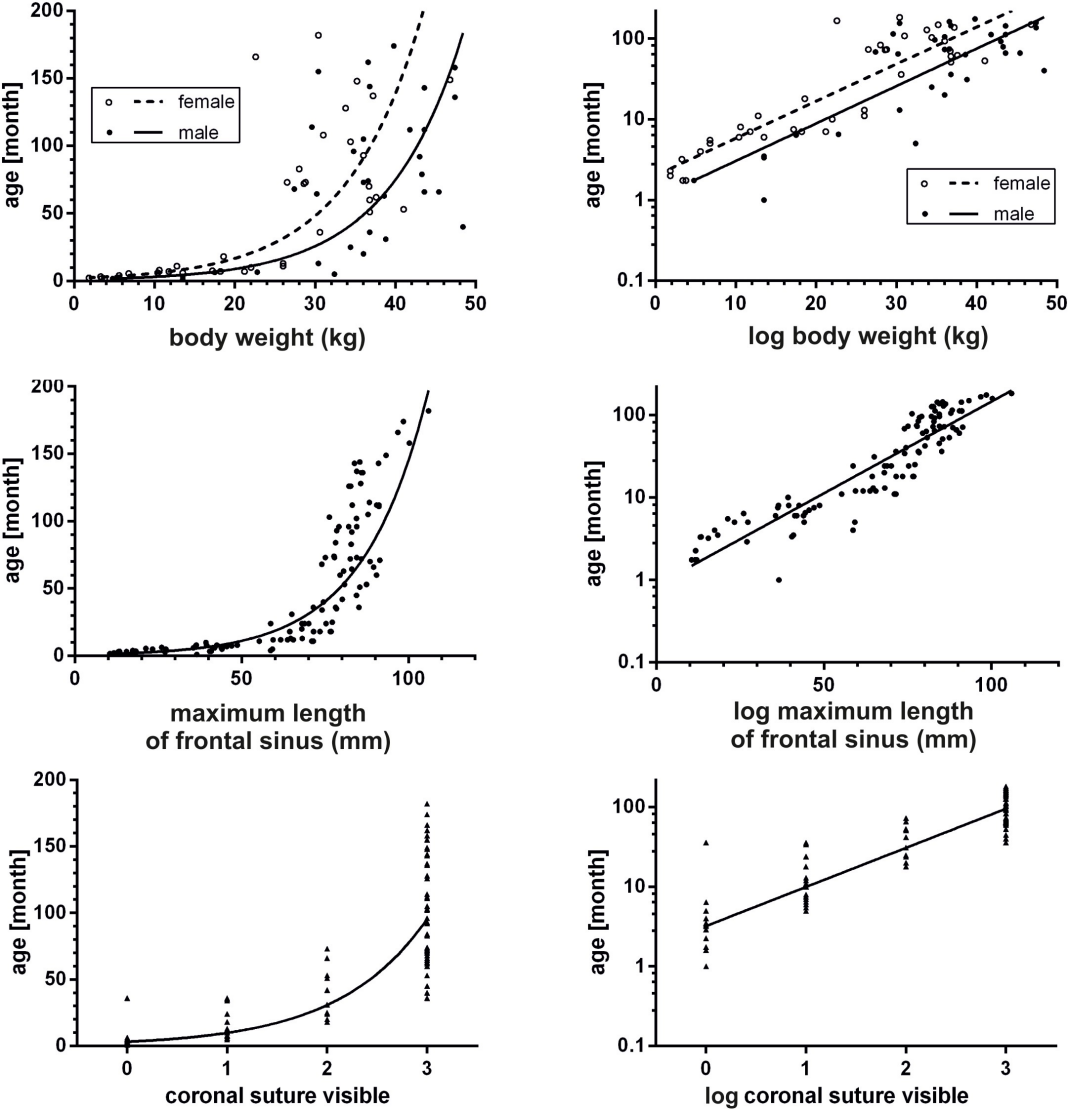
Correlation of measured variables with age. Graphic representation of the variable body weight, maximum length of frontal sinus and ossification status of the coronal suture in correlation with age in non-logarithmized and logarithmized form.

**Fig 11:**
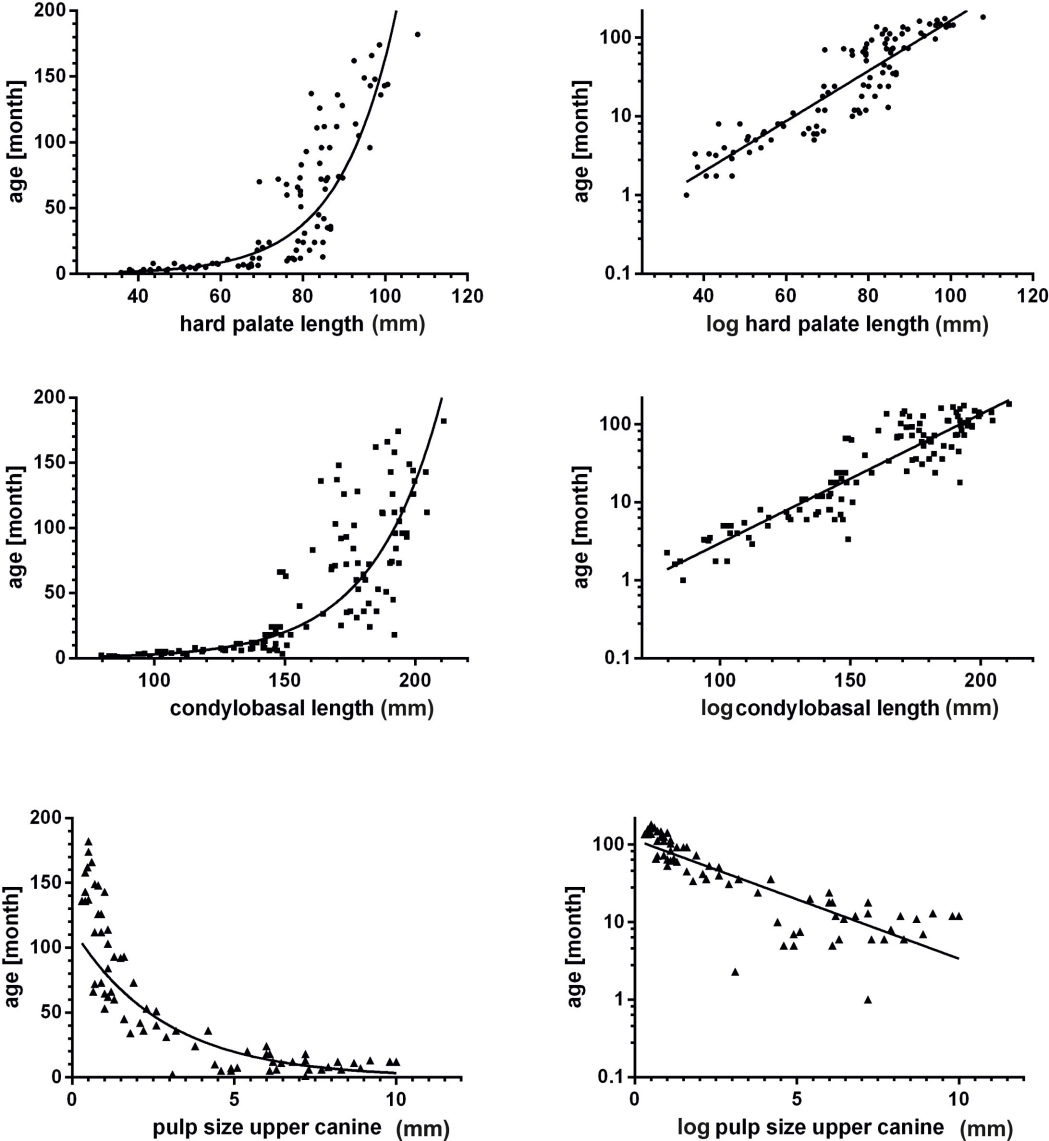
Correlation of measured variables with age. Graphic representation of the variable hard palate length, condylobasal length and pulp size of the upper canine in correlation with age in non-logarithmized and logarithmized form.

Results of the first step of the regression analyses (raw correlation) are summarized in Table 2. All parameters showed a global dependency on age. Most metric parameters showed an increase until around 150 months (12.5 years) (Figs 10 and 11). Sex only had an influence on the parameter body weight (p<0.001), but not on the other measured parameters. Therefore, both males and females were pooled in the analysis of age prediction. A complete set of measurements was obtained from 50 animals.

**Table 2:** Results of the regression analysis for each measured variable.

Variable	numbers	Coefficient of determination (r^2^)	F-value	p-value
Accessory carpal apophysis	41	0.4408	30.746	< 0.0001
Bodyweight kg	74	0.7099	176.178	< 0.0001
Coronal suture visible 0–3	121	0.8649	761.567	< 0.0001
C1 max lamina height	107	0.125	14.994	0.0002
C2 caudal physis	93	0.778	318.967	< 0.0001
C2 minimum vertebral canal height	83	0.7453	237.08	< 0.0001
C4 cranial physis	72	0.5369	81.148	< 0.0001
C4 caudal physis	70	0.7035	161.31	< 0.0001
Calcaneus apophysis	16	0.8436	75.496	< 0.0001
Clavicle visibility	48	0.3005	19.757	0.0001
Condylobasal length (cbl)	120	0.8342	593.84	< 0.0001
Cranial length (cl)	119	0.8158	518.044	< 0.0001
Cranial width	51	0.4927	47.598	< 0.0001
Distal femoral physis	34	0.8281	154.124	< 0.0001
Distal tibial physis	19	0.8412	90.47	< 0.0001
Distal humerus physis	41	0.6151	62.313	< 0.0001
Distal fibular physis	13	0.8595	67.314	< 0.0001
Distal radius physis	42	0.401	26.774	< 0.0001
Distal ulnar physis	41	0.3687	22.775	< 0.0001
Facial length (fl)	119	0.7765	406.492	< 0.0001
Frontal sinus index	111	0.1437	18.291	< 0.0001
Femoral head physis	34	0.8331	159.762	< 0.0001
Hard palate length (hpl)	98	0.8231	446.69	< 0.0001
Intercentrum 2 of axis fused	106	0.5574	130.963	< 0.0001
L5 cranial physis closure	65	0.535	72.48	< 0.0001
L5 caudal physis	65	0.747	185.969	< 0.0001
Maximum length of the frontal sinus (mlfs)	112	0.8449	599.3	< 0.0001
Maximum height of the frontal sinus	111	0.8059	452.679	< 0.0001
Major trochanter apophysis	33	0.8649	198.512	< 0.0001
Medial epicondyle apophysis	41	0.5916	56.496	< 0.0001
Metacarpal physis	24	0.3161	10.17	0.0042
Occipital height	97	0.8196	431.63	< 0.0001
Olecranon apophysis	44	0.4849	39.541	< 0.0001
Pulp size upper canine	72	0.6999	163.234	< 0.0001
Proximal tibial physis	32	0.7909	113.503	< 0.0001
Proximal radius physis	45	0.6005	64.632	< 0.0001
Patella visible	31	0.3388	14.857	0.0006
Proximal humers physis	55	0.7834	191.713	< 0.0001
Skull width	51	0.5568	61.568	< 0.0001
Skull length	120	0.6864	258.221	< 0.0001
Supraglenoid apophysis closed	61	0.8054	244.141	< 0.0001
T majus developed	58	0.8531	325.16	< 0.0001
Tibial crest apophysis	25	0.7865	84.716	< 0.0001

Multiple stepwise regression analysis revealed, as a first step, results of the following parameters: Closure status of the coronal suture status (cors) (f-value: 761.6; r^2^: 0.865); maximum length of the frontal sinus (mfsl) (f-value 599.3; r^2^: 0.845); condylobasal length (cbl) (f-value: 593.8; r^2^: 0.834); hard palate length (hpl) (f-value: 446.7; r^2^: 0.823); and facial length (facl) (f-value: 406.5; r^2^: 0.777). These parameters were most strongly correlated with age (p<0.0001 in each case; raw correlation; n = 94 observations or more). The addition of other parameters showed no significant improvement of the goodness of fit to the model. Taking these predictor variables and their regression coefficients into account, a regression equation for the relation between age and measured parameters was elaborated. The best model predicted age as:

lg(age)=0.0052xmfsl+0.0058xfacl+0.00278xcbl+0.1829xcors+0.0051xhpl–0.407.

**mfsl**: maximum length of the frontal sinus; **facl**: facial length; **cbl**: condylobasal length; **cors**: coronal suture status; **hpl**: hard palate length;

Multiple coefficient of determination for this equation was r^2 =^ 0.9581 with a standard error of estimation of 0.1236 (dispersion factor). In a non-logarithmised model, the following formula can be applied: Estimated age in months:

$$Age=10^{0.0052\;x\;mfsl+0.0058\;x\;facl+0.00278\;x\;cbl+0.1829\;x\;cors+0.0051\;x\;hpl–0.407}$$

**Fig 12:**
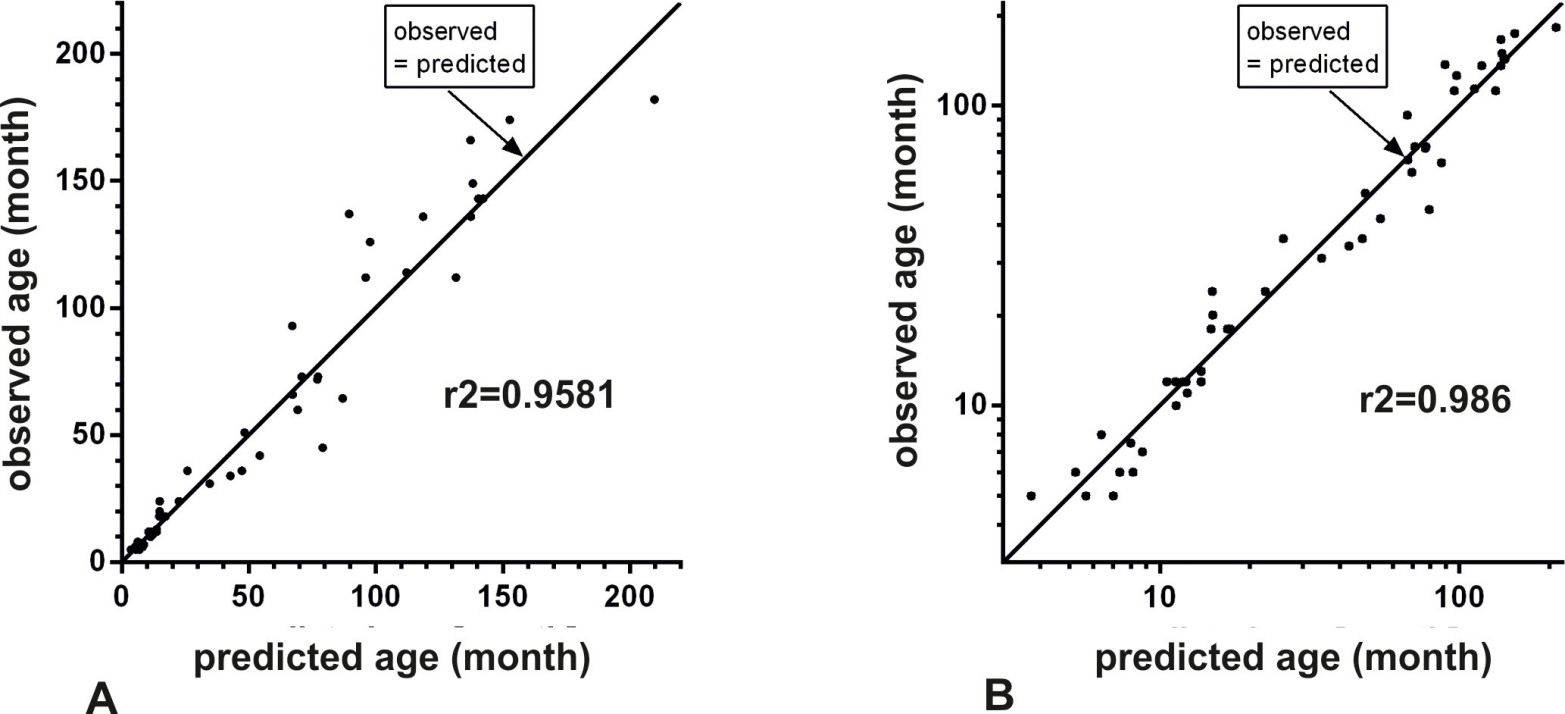
Normal probability plot for the residuals of predicted vs. observed age. Comparison of the regression of the predicted vs. the observed age and the measured coefficient of determination (r^2^) without (A) and with inclusion of the variable “pulp size upper canine” (B).

**mfsl**: maximum length of the frontal sinus; **facl**: facial length; **cbl**: condylobasal length; **cors**: coronal suture status; **hpl**: hard palate length;

The non-logarithmized form of the standard error of estimation is given by the dispersion factor of the estimation = 1.33 (Fig 12A). This corresponded to an approximate standard deviation for the age estimation of 33%.The consideration of the variable pulp size upper canine (pulpuc) and of quadratic forms of the best predictors resulted in an improved formula:

$$Age:\;10^{\left(0.0124\cdot mfsl+0.00414\cdot cbl+0.0594\cdot cors+0.0067\cdot hpl\;‐0.1634\cdot pulpuc‐0.00005855\cdot mfsl2+0.0110\cdot pulpuc2–0.03084\right)}$$

**mfsl**: maximum length of the frontal sinus; **cbl**: condylobasal length; **cors**: coronal suture status; **hpl**: hard palate length; **pulpuc**: Pulp size upper canine

This formula resulted in a multiple coefficient of determination of r^2^ = 0.968 and a standard error of the prediction (logarithmic version) of 0.0984. In the anti-logarithmic form this corresponded to a dispersion factor of 1.254 indicating a standard deviation of approximately 25.4% for the age determination (Fig 12 B). Here n = 50 observations were usable.

### Interobserver precision

**Fig 13:**
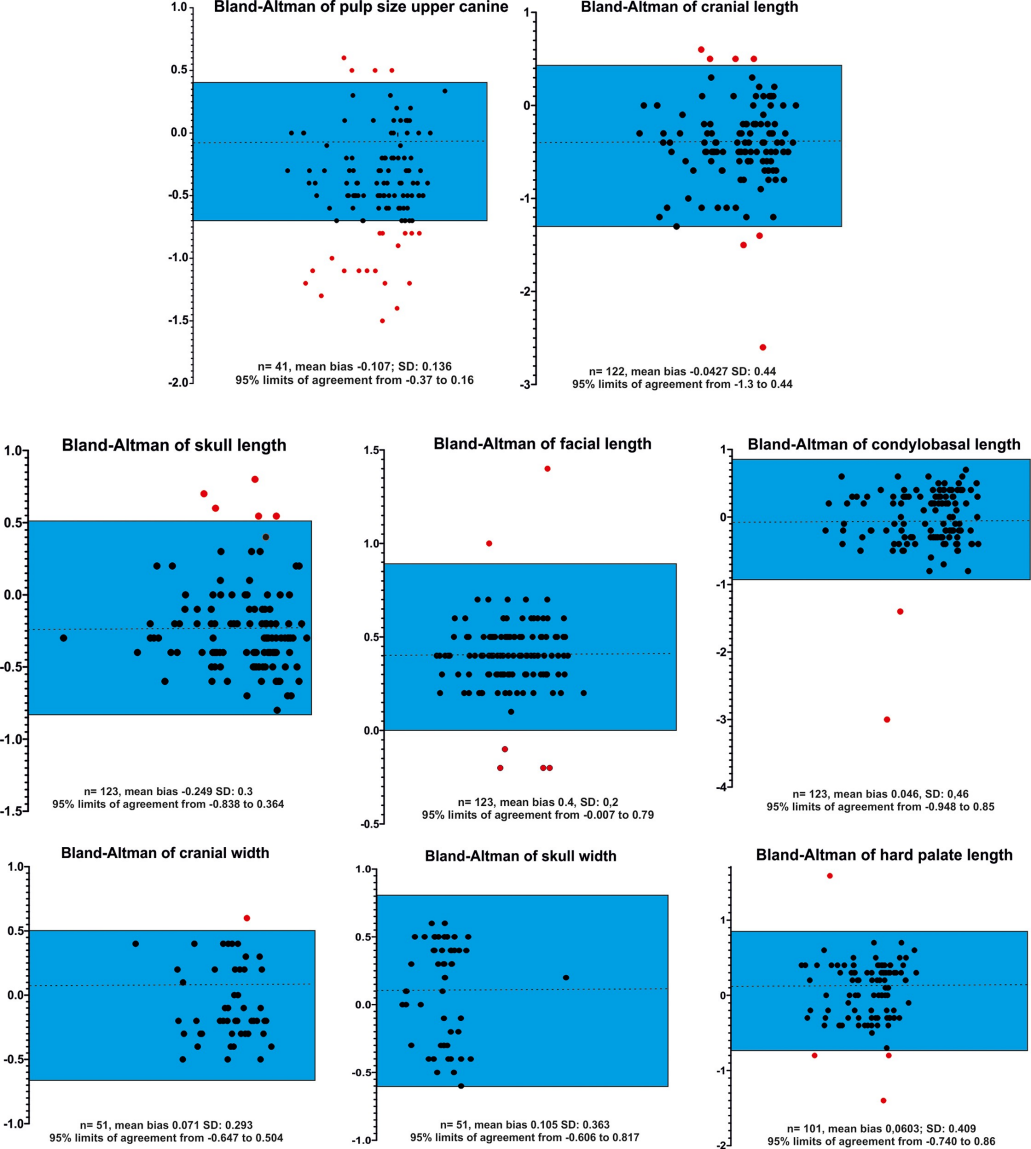
Bland Altman plots demonstrating interobserver variability of measurements. Graphical presentation of comparison of two measurements of different cranial and dental parameters (pulp size, cranial length, skull length, facial length, condylobasal length cranial width, skull width, hard palate length) in Bland-Altman plots. The reproducibility of measurements of two observers is demonstrated. The differences between the two measurements (black dots) are plotted against the averages of the differences. The blue box indicate the lower and upper limits of agreement (mean difference ± 2x standard deviations). 95% of all differences were within two standard deviations, representing excellent reproducibility. Outliers are marked as a red dot.

**Fig 14:**
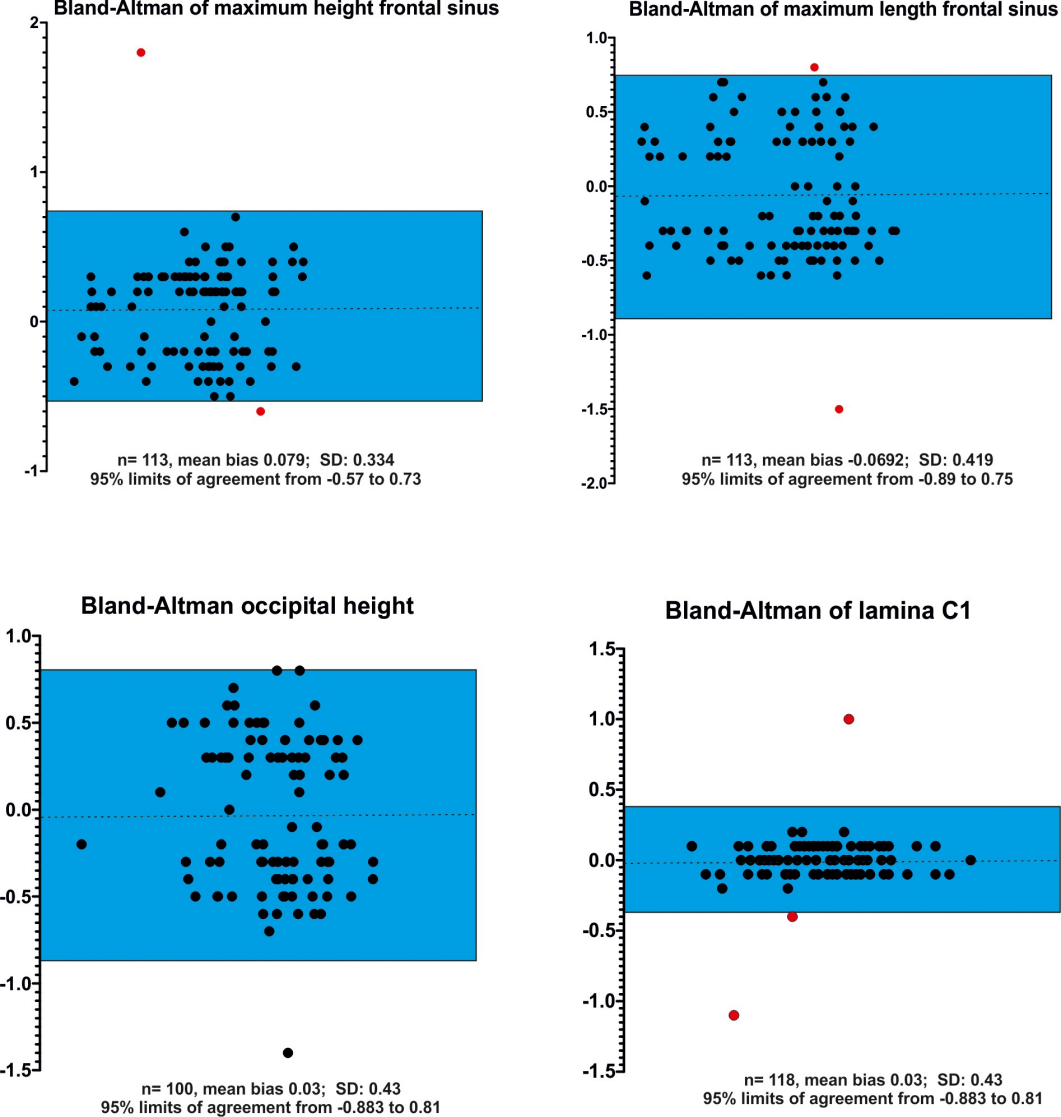
Bland Altman plots demonstrating interobserver variability of measurements. Graphical presentation of comparison of two measurements of different cranial and vertebral parameters (maximum height of the frontal sinus, maximum length of the frontal sinus, occipital height, maximum height of lamina C1) in Bland-Altman plots. The reproducibility of measurements of two observers is demonstrated. The differences between the two measurements (black dots) are plotted against the averages of the differences. The blue box indicate the lower and upper limits of agreement (mean difference ± 2x standard deviations). 95% of all differences were within two standard deviations, representing excellent reproducibility. Outliers are marked as a red dot.

Kappa statistics revealed excellent results for all ordinal parameters (closed vs. open). The assessment of reproducibility of numerical variables is shown in Figs 13 and 14. Ninety-five percent of the differences between the first and second measurements were less than ± 2 SD from the mean difference for all variables demonstrating good agreement between measurements except for pulp size of the upper canines. The mean difference in these calculations was negative, indicating a tendency to measure higher values of the second observer (MJS).

## Discussion

Prior to this study, information regarding fairly specific age estimation of living cheetahs was not available. This is the first attempt to provide a non-invasive methodology to estimate the age of cheetahs based on cranial and post-cranial skeletal features seen in radiographs of a large group of animals with a broad representation from birth to old age. Using a multiple stepwise regression model, we identified morphological parameters that can be used to approximate the age of cheetahs. The main advantage of the present study is the knowledge of the age of the examined animals at the time of radiographic examination. Marker and Dickman presented a collection of external morphological features of wild cheetahs that allowed age approximation [46]. However, the cheetahs examined in that study were assigned to 8 age groups each with a span of 6 months, which is not very specific. Furthermore, the age was only estimated in free-ranging animals based on body weight, social grouping and reproductive status.

Parameters reflecting linear skull growth were most significantly correlated with age in our model. Age determination studies across a wide range of species found a high relationship between age and linear skull measurements [10, 31–34]. Growth curves and equations from these relationships could be reliably used to estimate unknown age in other large felids [10]. Skull examinations have the advantage that factors that may negatively influence general skeletal growth seem to have less impact on the growth of the skull [48]. The skull, and especially the facial bones, were found to expand continuously throughout life in humans and other mammals including felids [20, 30, 38, 49]. This is in agreement with our study, in which craniometric parameters increased beyond sexual maturity [50] until they reached a plateau at 150 months. The width and height of the calvaria did not correlate with age to the same extent. The trend that cranial width and skull width do not show the same allometric increase with bodyweight and other measurements, and can even show a negative allometric growth, has been observed in other felids [33, 34].

The cheetah has a very distinct skull shape, which differs from the general pattern of felid skulls [46]. Cheetahs show characteristically inflated frontal bones with expanded frontal sinuses, which alleviate the high oxygen demand during high-speed chases [46, 51]. The longitudinal expansion of the frontal sinus correlated well with age. Frontal sinus size can be utilized to accurately predict the unknown age of humans [40] and other animals [41]. In humans, it reaches 95% of adult size at the end of puberty when general body and skull growth is almost completed, but continues beyond that time [42], which was also found in the cheetah.

The sutures of the neurocranium are generally open early in life and gradually close as maturity is attained. From all skull sutures, we analyzed the timing of coronal suture closure, because it can reliably be identified in radiographs and closure occurs along the entire suture at the same time and not partially like in other skull sutures [52]. It was found in some investigations that skull suture closure does not reflect ontogeny alone, but also cranial biomechanics relating to feeding behaviors [53]. However, the influence of muscle strain on the timing of coronal suture closure can be neglected, as the suture is not an insertion point of masticatory muscles like for example the sagittal suture [53, 54]. The closure level of this growth center seems to be mainly influenced by age explaining the strong correlation in our model.

In contrast to other studies, we could not find a strong relationship between dental pulp cavity size and age. Nevertheless the addition of the variable “pulp size” resulted in less dispersion and a more reliable age prediction, thus still giving it some value in our study. Radiography was proven to be suitable for pulp size measurement even in smaller species as, for example, the domestic cat [13, 17]. Measurement of pulp size significantly varied between observers in our study. The head radiographs used here were not specifically made as dental radiographs, which probably created some methodological bias. A prospective study evaluating pulp size under ideal dental radiographic conditions is likely to generate more precise data.

Elongation of long bones is driven by chondrogenesis at the physeal plate. Proliferation, hypertrophy, and differentiation of chondrocytes is regulated by an intricate network of endocrine signals, including growth hormone, insulin-like growth factor I, glucocorticoids, thyroid hormones, sexual hormones, vitamin D, and leptin [55]. At the beginning of puberty, rising levels of androgens and estrogen induce the stimulation of the growth hormone and insuline-like growth factor to produce a growth spurt [55]. At the end of puberty initiation of closure is promoted by high levels of sex hormones that activate or deactivate parathyroid hormone–related protein, Indian hedgehog gene, fibroblast growth factor, bone morphogenetic protein, and other signaling pathways in both basi-cranial and long-bone development [56]. However, corresponding to general growth, physeal closure and ossification are dependent not only on intrinsic but also on extrinsic factors such as diet. Our study was conducted with captive and semi-captive cheetahs from different locations and diets that may have differed from natural ones, which may give rise to possible nutritional biases. Some studies on large felids revealed that captivity status has in fact a higher impact on morphological variation than sexual dimorphism [15, 16]. Captive cheetahs can be on a higher nutritional level compared to free-ranging animals [57, 58] and confounding influences of calorie intake, or dietary supplements on physeal fusion must be considered [10]. This might be an explanation for the fact, that morphological features measured in the axial and appendicular skeleton were not correlated enough to contribute to the best predictive age model equation. Specifically, the timing of physeal closure is dependent upon anatomical site, hormonal status, caloric supply and dietary composition [59]. As many cheetahs were involved in breeding programs, influence from an altered hormonal status can be ruled out. The influence of sex on growth plate closure was not significant in our study, which made diet the most likely bias. Deficiencies of fat-soluble vitamins, especially vitamin A, can have a severe impact on general health and, relevant to our study, endochondral bone growth. Vitamin A deficiency can occur even under controlled and optimized feeding strategies [58]. Several studies have shown that Vitamin A stimulates osteoblast differentiation, as well as promote osteogenesis in the physes of the long bones and cranial base. Both, vitamin A deficiency and excess can therefore cause accelerated or delayed closure of the physes [60, 61]. Inadequate intake of vitamin-D may also severely influence duration of growth of long bones and other skeletal parts. However, cheetahs with radiological evidence of metabolic bone conditions were excluded from the investigation. In general, we found that closure of the physes in the appendicular skeleton of cheetahs occurs later in life compared to dogs and cats, in which the appendicular physes are usually closed by 12–15 months [40, 41, 62, 63]. We documented appendicular physeal closure to occur between 18 and 31 months. Male and female cheetahs attain sexual and physical maturity at about 21 months [47], which generally corresponds to the physeal closure times and cessation of body weight increases around 20 months as found in our study. These findings correspond to the data found in investigations of the appendicular skeleton in lions [64, 65].

The present investigation provides the best currently available equation for age estimation in cheetahs. However, the standard error in our estimation was 25%, which could produce some error. In felids, absolute size and shape change are not always correlated with time of development. Some individuals may attain great changes in less time than others [33]. Although the cheetah was reported to be the most morphologically consistent felid [66, 67], morphological variation within age classes may blur the distinctions between ages, which can be based on geographic, sexual and individual variation [68–72]. African subspecies of cheetahs can show considerable variation in body size [67] that can be related to resource availability, but might also represent a splitting of populations into subgroups with distinct skeletal features and dimensions. To date this has not been well investigated in the cheetah. However, as only South African cheetahs were included into the study, bias from geographical variation can be ruled out.

Sex had no significant influence on the values in this model. This is interesting because sexual dimorphism is widespread among carnivorans and pronounced in most wild felids. A number of studies reported that body weight can be safely used to distinguish between male and female individuals [68–72]. However, sexual dimorphism is not equally pronounced in all felids. There is only a small degree of sexual dimorphism between the sexes in *Acinonyx* species. This monomorphism seems to be quite unique to the cheetah [67]. Male cheetahs can be larger than females in regards to their body mass, body length, and chest girth, which seems to be more evident in animals older than 12 months [67, 73]. However, they are by far less dimorphic concerning cranial and dental features [74, 75]. Felids that live in larger groups containing a dominant male show higher amounts of sexual dimorphism than solitary species and do not engage in intense competition for females, which is the case in cheetahs [76]. Inbreeding and genetic monomorphism of the cheetah were also proposed for the finding that sexual dimorphism is extremely limited in this species [77].

Individual variation may relate to variation of ontogenetic development. Recorded birth weights of cubs vary a great deal and postnatal growth rate is dependent on various factors, such as litter size, gestation length, amount of inbreeding, parity of the dam, and sex of the cub. The birth weight of wild cheetah cubs was markedly lower than the birth weight of captive cheetahs [66–68, 78–80]. Morphological analyses of the skull of South African cheetahs revealed that cranial-, and to a lesser extent odontometric measurements tend to show a higher asymmetry relative to the other large cats [75, 76]. This loss of morphological stability was attributed to genetic monomorphism based on a severe evolutionary bottleneck followed by inbreeding in the history of the cheetah [77]. Deviations from phenotypical symmetry (“fluctuating asymmetry”) among inbred and genetically depauperate species are thought to reflect the loss of the ability of individuals or populations to resist epigenetic influences on skull ontogeny [74, 75]. Whether such fluctuating asymmetry on the cheetah really exists [81–84] and whether it has an influence on our measurements remains inconclusive.

Some factors limit the scope and applicability of the present investigation as most parameters examined in this investigation have elements of subjectivity or bias. The retrospective nature of the study, using clinical case material without standardization in radiographic positioning and protocols, is the main limitation. The comparison of our estimates with those of other studies show that these problems probably do not bias our analyses in a consistent way, but may have an influence on some measurements [64]. Finally, a complete radiographic study, displaying all body parts of interest was not available in all animals. There were less appendicular skeleton radiographs than skull radiographs, which could have influenced our regression model.

## Conclusion

The status of closure of the coronal suture, the maximum length of the frontal sinus, the condylobasal-, hard palate, and facial length are most significantly correlated with age. Together with the pulp size of the upper canine, these values can be used for an age approximation in cheetahs.

## Supporting information

S1 Table Measured parameters in cheetah skull and skeleton. (XLSX)

S1 Formula Ready to use formula for age determination. (XLSX)
